# EEBR induces Caspase‐1‐dependent pyroptosis through the NF‐κB/NLRP3 signalling cascade in non‐small cell lung cancer

**DOI:** 10.1111/jcmm.18094

**Published:** 2024-01-12

**Authors:** Xin Zhao, Chao Chen, Weina Han, Min Liang, Yuanyuan Cheng, Yingjie Chen, Defeng Pang, Haoqi Lei, Xuefei Feng, Shifeng Cao, Zhixiong Li, Jinhui Wang, Yan Zhang, Baofeng Yang

**Affiliations:** ^1^ Department of Pharmacology (State‐Province Key Laboratories of Biomedicine‐Pharmaceutics of China, Key Laboratory of Cardiovascular Medicine Research, Ministry of Education), College of Pharmacy Harbin Medical University Harbin China; ^2^ National Key Laboratory of Frigid Zone Cardiovascular Diseases (NKLFZCD) Harbin China; ^3^ Department of Medicinal Chemistry and Natural Medicine Chemistry, College of Pharmacy Harbin Medical University Harbin Heilongjiang China; ^4^ Research Unit of Noninfectious Chronic Diseases in Frigid Zone, 2019RU070 Chinese Academy of Medical Sciences Harbin China

**Keywords:** Caspase‐1, EEBR, GSDMD, NF‐κB, NSCLC, pyroptosis

## Abstract

Lung cancer is a leading cause of cancer‐related deaths worldwide. Recent studies have identified pyroptosis, a type of programmed cell death, as a critical process in the development and progression of lung cancer. In this study, we investigated the effect of EEBR, a new compound synthesized by our team, on pyroptosis in non‐small cell lung cancer cells (NSCLC) and the underlying molecular mechanisms. Our results demonstrated that EEBR significantly reduced the proliferation and metastasis of NSCLC cells in vitro. Moreover, EEBR‐induced pyroptosis in NSCLC cells, as evidenced by cell membrane rupture, the release of cytokines such as interleukin‐18 and interleukin‐1 beta and the promotion of Gasdermin D cleavage in a Caspase‐1‐dependent manner. Furthermore, EEBR promoted the nuclear translocation of NF‐κB and upregulated the protein level of NLRP3. Subsequent studies revealed that EEBR‐induced pyroptosis was suppressed by the inhibition of NF‐κB. Finally, EEBR effectively suppressed the growth of lung cancer xenograft tumours by promoting NSCLC pyroptosis in animal models. Taken together, our findings suggest that EEBR induces Caspase‐1‐dependent pyroptosis through the NF‐κB/NLRP3 signalling cascade in NSCLC, highlighting its potential as a candidate drug for NSCLC treatment.

## 1 INTRODUCTION

Lung cancer is a major public health concern and the leading cause of cancer deaths worldwide, responsible for approximately 1.8 million deaths annually.^1^, ^2^ Small cell lung cancer (SCLC) and non‐small cell lung cancer (NSCLC) represent the predominant subtypes of lung cancer, constituting 15% and 85% of all cases, respectively. NSCLC is comprised of three primary histological subcategories: squamous cell carcinoma (SCC), adenocarcinoma (AD) and large cell carcinoma (LCC). Unfortunately, the majority of patients receive a diagnosis during the intermediate or advanced stages of the disease, resulting in suboptimal treatment outcomes. As a result, the 5‐year survival rate is less than 5%.^3^ In addition to surgery, primary treatment methods for lung cancer include chemotherapy utilizing cisplatin (DDP) and other medications, immunotherapy utilizing PD‐1/PD‐L1 and targeted therapy utilizing EGFR inhibitors. It is noteworthy, however, that prolonged use of most of these treatments results in serious adverse reactions or drug resistance development.

The goal of treating cancer has always been to eliminate malignant cells. While most drugs impede tumour development by promoting apoptosis, research has shown that cisplatin or tyrosine kinase inhibitors (TKIs) may also stimulate pyroptosis in cancer cells.^4^, ^5^ Pyroptosis is a form of programmed cell death mediated by the Gasdermin family and involves continuous cellular distension until the cell membrane ruptures, resulting in the release of cellular contents and the initiation of a hyperinflammatory response.^6^ Pyroptosis can be induced by activation of the Caspase‐1 or caspase‐4/5/11 pathways, which cleave Gasdermin D (GSDMD) proteins and perforate N‐terminal domain of GSDMD (N‐GSDMD) oligomers on the cell membrane.^7^, ^8^, ^9^ Pyroptosis is associated with various diseases, including infectious, neurological and atherosclerotic diseases.^10^ Recent evidence suggests that pyroptosis plays a crucial role in inhibiting tumour cell proliferation in vitro and tumour growth in vivo, making it a potential therapeutic target for the treatment of various cancers.

9‐(2‐ethoxyethoxy)‐10‐methoxy‐5,6‐dihydro‐[1,3]dioxolo[4,5‐g]isoquinolino[3,2‐a]isoquinolin‐7‐ium bromide (EEBR) is a compound synthesized by our team. It is an alkaloid that contains quaternary nitrogen basic skeleton. Previous research has shown that quaternary nitrogen is an important chemical group with the pharmacological activity of anti‐tumour.^11^ Therefore, we designed and synthetized EEBR with the basic skeleton of alkaloid quaternary nitrogen. To improve the solubility of the molecule in water and organic solvents and to facilitate binding to functional proteins, an ethyl ether substituent was introduced, which also increased the molecule's flexibility and is expected to increase its anti‐tumour activity. However, whether EEBR prevents the advancement of NSCLC and the related molecular mechanisms have not been revealed.

Two of the hallmarks of tumour cells are infinite proliferation and resistance to cell death that can be induced by various factors. Therefore, inducing tumour cell death through various means is a fundamental strategy for non‐surgical tumour treatment. Recent studies have revealed that various TKIs and loplatin can induce cell death through pyroptosis associated with certain tumours.^12^, ^13^, ^14^ Pyroptosis has shown promise with its rapid and broad tumour‐killing effect, potentially offering new ideas for tumour treatment. In this study, we demonstrated that the novel compound EEBR possesses the ability to significantly reduce the proliferation and metastasis of A549 cells in vitro by inducing pyroptosis. EEBR also suppressed the growth of NSCLC xenograft tumours in vivo. Mechanistically, EEBR activated the nuclear factor kappa‐light‐chain‐enhancer of activated B cells (NF‐κB)/NOD‐like receptor 3 (NLRP3) signalling pathways and promoted GSDMD cleavage in a Caspase‐1‐dependent manner, ultimately leading to pyroptosis induction in NSCLC both in vitro and in vivo.

## 2 MATERIALS AND METHODS

### 2.1 Cells, animals and reagents

The A549 and H1299 human lung cancer cells were provided by the Pharmacology Laboratory of Harbin Medical University, and the most recent STR identification was in September 2023 (Figure S1A,B). BALB/c Nude mice were purchased from Beijing Viton Lihua Laboratory Animal Technology Co. The experimental compound EEBR was provided by the Laboratory of Natural Drugs and Medicinal Chemistry, Harbin Medical University, while cisplatin was obtained from Qilu Pharmaceutical Co., LTD. VX‐765 and BAY 11‐7082 inhibitors were purchased from Dalian Meilun Biotechnology Co., LTD. Trypsin, 1640 medium and penicillin/streptomycin were supplied by Gibco (Carlsbad, CA, USA), while fetal bovine serum (FBS) was obtained from Clark Corporation, USA. The Cell Counting Kit‐8 (CCK8) was purchased from Dojindo and the BCA protein quantitation kit from Beyotime. The antibodies specific for NF‐κB (cat# A10609), NLRP3 (cat# A5652), cleaved Caspase‐1 (cat# A23429) and GSDMD (cat# A20728) were purchased from ABclonal; N‐terminal GSDMD (cat# ab215203) was purchased from Abcam; β‐actin (cat# bs‐0061R), Caspase‐1 (cat# bs‐10442R), interleukin‐18 (IL‐18) (cat# bs‐4986R), interleukin‐1 beta (IL‐1β) (cat# bs‐25615R) antibodies were obtained from Bioss.

### 2.2 CCK8 assay

Cell viability was assessed using the CCK8 (Dojindo, Shanghai, China). A549 and H1299 cells were seeded at a density of 5 × 10^3^ cells per well in 96‐well plates with 5 × 10^3^ cells per well and treated with different concentrations of EEBR and DDP. After 24 h of incubation, 10 μL CCK8 solution was added to each well and the plates were incubated for 1 h at 37°C in the dark. The optical density (OD) value was then measured at 450 nm using a multifunctional microplate reader (Bio Tek, U.S.).

### 2.3 Transwell assay

Transwell assays were performed using Transwell chambers with 8 μm pore size (Corning). First, the chambers were coated and solidified with matrigel. A549 and H1299 cells were resuspended in 200 μL of serum‐free 1640 medium at a density of 5 × 10^4^ cells/well and added to the upper chamber of the Transwell cell culture chambers. The lower chamber was filled with DDP prepared from 500 μL of complete medium and various concentrations of EEBR. The cells were then incubated at 37°C in a humidified incubator containing 5% CO_2_ for 24 h. After incubation, the cells were fixed with 4% formaldehyde for 30 min at room temperature and stained with 0.1% crystal violet (Aladdin, Shanghai). The cells were then imaged using an inverted microscope.

### 2.4 Wound healing assay

After the confluence of A549 cells reached 90%, a gentle line was drawn inside each well followed by two washes with PBS to remove floating cells. Then, each experimental group was treated with serum‐free culture solution, DDP and various concentrations of EEBR before incubating for 24 h. The cell migration of each subgroup was recorded at 0, 12 and 24 h by randomly capturing images using a microscope. The images were analysed using Image Pro Plus software.

### 2.5 Transmission electron microscopy

Transmission electron microscopy experiments were conducted in collaboration with the Pathology Department of Harbin Medical University. Briefly, A549 and H1299 cells were seeded onto 6‐well plates at a density of 3 × 10^5^ cells/well and treated with cisplatin and EEBR at concentrations of 10, 30 and 100 μM for 24 h. After discarding the culture medium, cells were washed with 1 × PBS and fixed with a solution of 4% paraformaldehyde and 2% glutaraldehyde for 1 h. The fixed cells were then post‐fixed in 1% osmium tetroxide and 0.5% potassium ferricyanide in cacodylate buffer for 1 h, and subsequently embedded in straight resin and cured at 80°C for 24 h. Finally, transmission electron microscopy (Thermo Fisher Scientific, Waltham, MA, USA) was used to analyse the samples.

### 2.6 Lactate dehydrogenase release assay

To assess the cytotoxicity of EEBR‐induced A549 and H1299 cells, lactate dehydrogenase (LDH) activity was determined using the LDH Assay Kit (Nanjing Jiancheng, Nanjing, China) according to the manufacturer's instructions. Briefly, the cells were seeded in 6‐well plates until they reached the desired confluency. After treatment with EEBR for 24 h, the supernatant was collected, and the absorbance value was measured at 450 nm. LDH activity was calculated using the following formula: LDH activity (U/L) = (assay OD − control OD)/(standard OD − blank OD) × standard (0.2 μmol/mL) × 1000.

### 2.7 Propidium iodide uptake assay

To confirm whether pyroptosis induced pore formation on cell membranes, staining was performed using the Hoechst 33342/propidium iodide (PI) Double Staining Kit (Nanjing Jiancheng, Nanjing, China) according to the manufacturer's instructions. Briefly, A549 and H1299 cells were seeded in 6‐well plates and dosed with the test compounds after reaching the appropriate cell density. After 24 h of dosing, cells were rinsed with PBS and collected. Then, 10 μL Heochst 33342 and 5 μL PI were added, mixed gently and incubated in room temperature and in the dark for 5–15 min. Finally, the cells with red and blue fluorescence were observed under a fluorescence microscope.

### 2.8 Western blot

Proteins were extracted from tissues or cells using RIPA lysate (Beyotime, Nanjing, China), and their contents were determined using the BCA protein quantitative kit (Beyotime, Nanjing, China). The protein samples were then subjected to sodium dodecyl sulphate‐polyacrylamide gel electrophoresis (Beyotime, Nanjing, China) and transferred onto NC membranes (Millipore, Germany). The membranes were blocked with 5% skim milk (Biofroxx, Germany) at room temperature for 2 h, followed by incubation with the primary antibody overnight at 4°C. After washing, the membranes were incubated with a secondary antibody, Goat Anti‐Rabbit or Mouse (Li‐COR, USA). Finally, the protein bands were visualized and analysed by scanning the membranes using Odyssey (Li‐COR, USA) to determine their grey value.

### 2.9 Enzyme‐linked immunospecific assay

To determine the levels of IL‐1β, IL‐18 and Caspase‐1 in tumour cells and tissues, enzyme‐linked immunospecific assay (ELISA) kits were used following the manufacturer's instructions. For in vitro experiments, culture media were collected immediately after treatment, and the supernatant was collected for testing after clearing the samples by centrifugation at 15,000 × g for 10 min. Standard curves were generated based on the kits' standards to ensure accurate quantification.

### 2.10 Quantitative real‐time RT‐PCR

In order to assess the impact of EEBR on NF‐κB expression, quantitative real‐time PCR (qRT‐PCR) amplification was conducted. Following a 24‐h treatment with EEBR (10, 30, 100 μM), cellular samples were collected and total RNA was extracted utilizing TRIzol® reagent (Invitrogen, USA). Equivalent quantities of RNA were subjected to reverse transcription into cDNA using the Revert Aid First Strand cDNA Synthesis Kit (Thermo Scientific, Boston, USA). Subsequently, the resulting cDNA was amplified using UltraSYBR Green qPCR Mixture (with ROX) according to the subsequent protocol: 10 min at 95 °C for the initial denaturation, followed by 95 °C for 15 s for 40 cycles, 60 °C for 1 min at the cycling stage and 95 °C for 15 s, 60 °C for 1 min, 95 °C for 15 s at the melt curve stage. Primer sequences are shown in below:

NF‐κB‐F: (5′‐AACAGAGAGGATTTCGTTTCCG‐3′)
NF‐κB‐R: (5′‐TTTGACCTGAGGGTAAGACTTCT‐3′).

### 2.11 Xenografts tumour model

A tumour cell suspension was prepared by adjusting A549 cells to 5 × 10^7^ cells/mL using filtered PBS. Subsequently, 0.3 mL of the tumour cell suspension was subcutaneously injected into the right back of each BALB/c Nude mouse. After successful in vitro transplantation of tumour construction, the mice were randomly assigned to the tumour model, DDP, 1, 3 and 10 mg/kg EEBR groups. DDP was intraperitoneally administered once every 2 days, while EEBR was administered by gavage daily. After 28 days of administration, the mice were euthanized, and the tumour size and weight were measured. Tumour volumes were calculated using the formula: volume = length × width^2^/2.

### 2.12 Haematoxylin and eosin staining

Fresh tissues were fixed in 10% formaldehyde solution, cleaned with PBS and embedded in paraffin wax to create 5 μm thick tissue sections. The paraffin sections were then graded and dehydrated using various ethanol concentrations and stained with haematoxylin and eosin. Finally, the sections were sealed with neutral resin, dried and observed under a light microscope. Photographs were taken for recording purposes.

### 2.13 Immunofluorescence

Tumour tissue sections were dewaxed and immersed in xylene for 3 min, followed by 100% ethanol, 85% ethanol, 75% ethanol and water for 5 min each. Antigen repair was performed by heating the sodium citrate repair solution (Beyotime, China) at 85°C for 25 min. The sections were then blocked with 5% BSA (Biotopped, China) for 30 min at room temperature before being incubated with the primary antibody overnight at 4°C. Next, a fluorescent secondary antibody (Goat anti‐rabbit or mouse Alexa Fluor 488; Li‐COR, USA) was applied to the sections and incubated for 1 h at room temperature. The nucleus was stained with DAPI (Biotopped, China) for 5 min. Between each step, the sections were washed with PBS for 15 min. Finally, the fluorescence images were captured using a confocal fluorescence microscope.

### 2.14 Statistical analysis

The data are expressed as mean ± standard error (SEM). Statistical analysis was performed using GraphPad Prism 8.0. One‐way analysis of variance (ANOVA) was used to compare multiple groups, while a *t*‐test was used for comparisons between two groups. *p* < 0.05 was considered statistically significant.

## 3 RESULTS

### 3.1 EEBR inhibits the proliferation, migration and invasion of NSCLC cells

**FIGURE 1:**
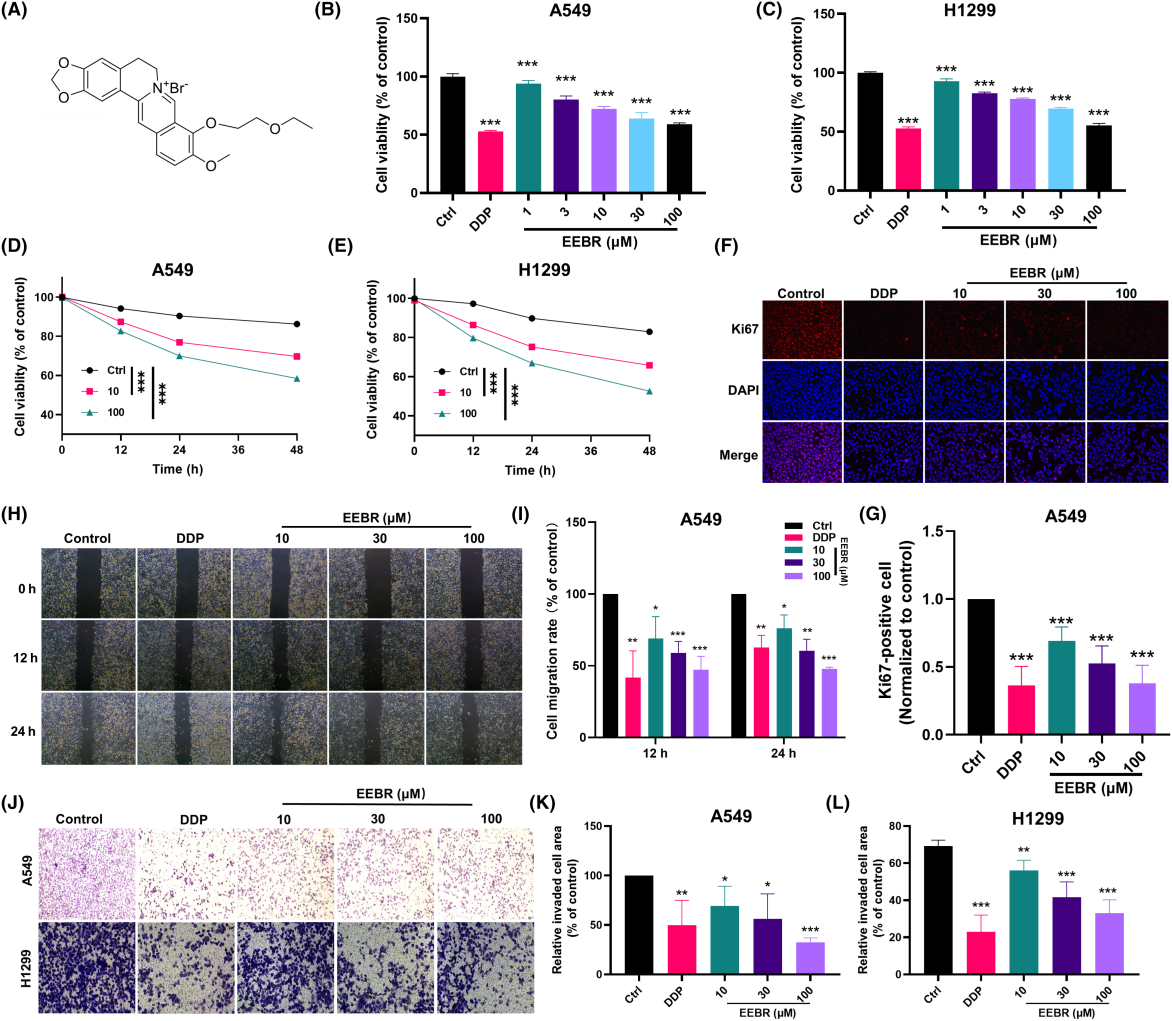
EEBR inhibits the proliferation, migration and invasion of NSCLC cells. (A) The structural formula of EEBR. (B, C) CCK8 assay showing the inhibition of the viability of A549 and H1299 cells by EEBR. (D, E) EEBR inhibits cell viability of A549 and H1299 in a time‐dependent manner. (F, G) Ki67 staining showing that EEBR decreased the rate of Ki67‐positive cells. (H, I) Wounding healing assay showing the inhibition of migration of A549 cells by EEBR. (J, L) Transwell assay showing that EEBR inhibited the invasion of A549 cells. The data are presented as the mean ± SEM. *p < 0.05; **p < 0.01; ***p < 0.001 versus control; *n* = 4.

We initially constructed EEBR and characterized the chemical structure using mass spectrometry (Figure 1A; Figure S1). To evaluate the response of A549 and H1299 cells to various doses of EEBR treatment, a CCK8 assay was performed to determine cell viability. The results revealed that EEBR inhibited human NSCLC cell growth in a dose and time‐dependent manner (Figure 1B–E). Immunofluorescence displayed that the proliferation markers Ki‐67 was generally expressed at a higher level in the control group, but its expression decreased upon exposure to EEBR and DDP (Figure 1F,G). Next, wound healing (Figure 1H,I) and Matrigel‐coated transwell invasion assays (Figure 1J–L) were used to assess cell migration and invasion, respectively. As expected, EEBR at variable concentrations inhibited cell migration and invasion in a dose‐dependent manner.

### 3.2 EEBR induces pyroptosis in NSCLC cells

**FIGURE 2:**
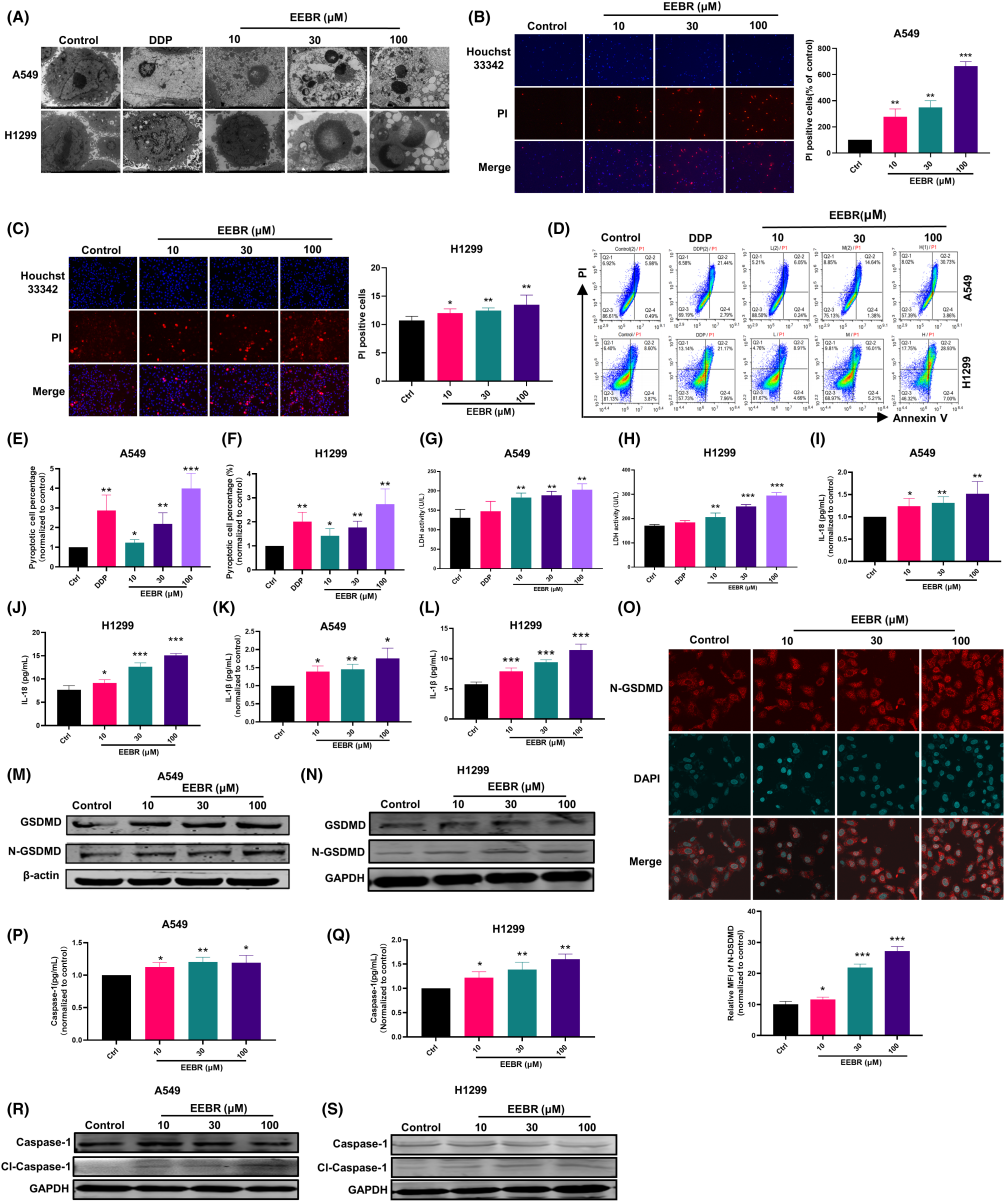
EEBR induces pyroptosis in NSCLC cells. (A) The ultrastructure ofA549 and H1299 cells observed under a transmission electron microscope. (B, C) Representative images of A549 (B) and H1299 (C) cells double stained by PI (red) displaying necrotic cells and Hoechst (blue) displaying apoptotic cells. (D–F) Flow cytometry analysis of Annexin V/PI double staining and pyroptosis cells in the Q2 area. (G, H) Effects of different concentrations of EEBR on LDH activity in A549 (G) and H1299 (H) cells. (I–L) The contents of IL‐18 (I, J) and IL‐1β (K, L) in cell supernatant determined by ELISA. (M, N) The expression of GSDMD and N‐GSDMD in A549 (M) and H1299 (N) cells with different concentrations of EEBR determined by western blot analysis. (O) A549 cells stained with immunofluorescence to detect N‐GSDMD with different concentrations of EEBR. (P, Q) The content of Caspase‐1 in A549 (P) and H1299 (Q) cells with different concentrations of EEBR quantified by ELISA kit. (R, S) The expression of Caspase‐1 and cleaved‐ Caspase‐1 in A549 (R) and H1299 (S) cells with different concentrations of EEBR determined by western blot analysis. The data are presented as the mean ± SEM. *p < 0.05; **p < 0.01; ***p < 0.001 versus control; *n* = 4.

To investigate the effect of EEBR on the cytological characteristics of A549 and H1299 cells, we performed transmission electron microscopy (TEM) analysis. As shown in Figure 2A, A549 and H1299 cells exposed to EEBR exhibited a ruptured morphology, which was not observed in cells incubated with only solvent or DDP. Moreover, we performed double staining with Hoechst 33342/PI to assess cell membrane rupture, where Hoechst 33342 stained the nuclei of cells, while PI penetrated dying cells with the loss of cell membrane integrity. The administration of EEBR results in the intracellular entry of PI, leading to an increase in PI‐positive cells (Figure 2B,C). Additionally, we used annexin V‐FITC/PI staining and flow cytometry to analyse A549 and H1299 cell death after treatment with EEBR for 24 h. Annexin V‐FITC/PI staining revealed that EEBR‐induced cell membrane rupture‐dependent death (Annexin V^+^/PI^+^; Figure 2D–F).

The visible impairment of the cell membrane let us to hypothesize that EEBR induces pyroptosis, a type of inflammation‐activated programmed cell death characterized by cell swelling, cell membrane rupture and release of cytoplasmic contents, which include LDH and pro‐inflammatory molecules such as IL‐1β and IL‐18.^7^ Accordingly, we observed an increase in LDH, IL‐1β and IL‐18 levels in the A549 and H1299 cell supernatant after EEBR treatment (Figure 2G–L).

GSDMD is a pore‐forming protein that plays a critical role in pyroptosis, and it is cleaved by Caspase‐1 to form micropores on the cell membrane through N‐GSDMD oligomerization.^15^, ^16^ We, therefore, investigated whether EEBR would activate GSDMD in A549 and H1299 cells. Immunoblotting results indicated that EEBR increased GSDMD expression and improved its cleavage (Figure 2M,N). Immunofluorescence staining against cleaved N‐GSDMD revealed that EEBR significantly increased N‐GSDMD expression compared to the control group, and it oligomerized on the cell membrane in A549 cells (Figure 2O). Emerging evidence suggested that cleavage of GSDMD proteins is mediated by Caspase‐1 and ultimately initiates pyroptosis. Consistently, ELISA showed that EEBR treatment resulted in a marked increase in Caspase‐1 levels (Figure 2P,Q). In addition, the expression of cleaved Caspase‐1 in A549 and H1299 cells was also increased due to EEBR treatment. Overall, these findings suggest that EEBR induces pyroptosis in A549 and H1299 cells.

### 3.3 EEBR induces Caspase‐1‐dependent GSDMD cleavage

**FIGURE 3:**
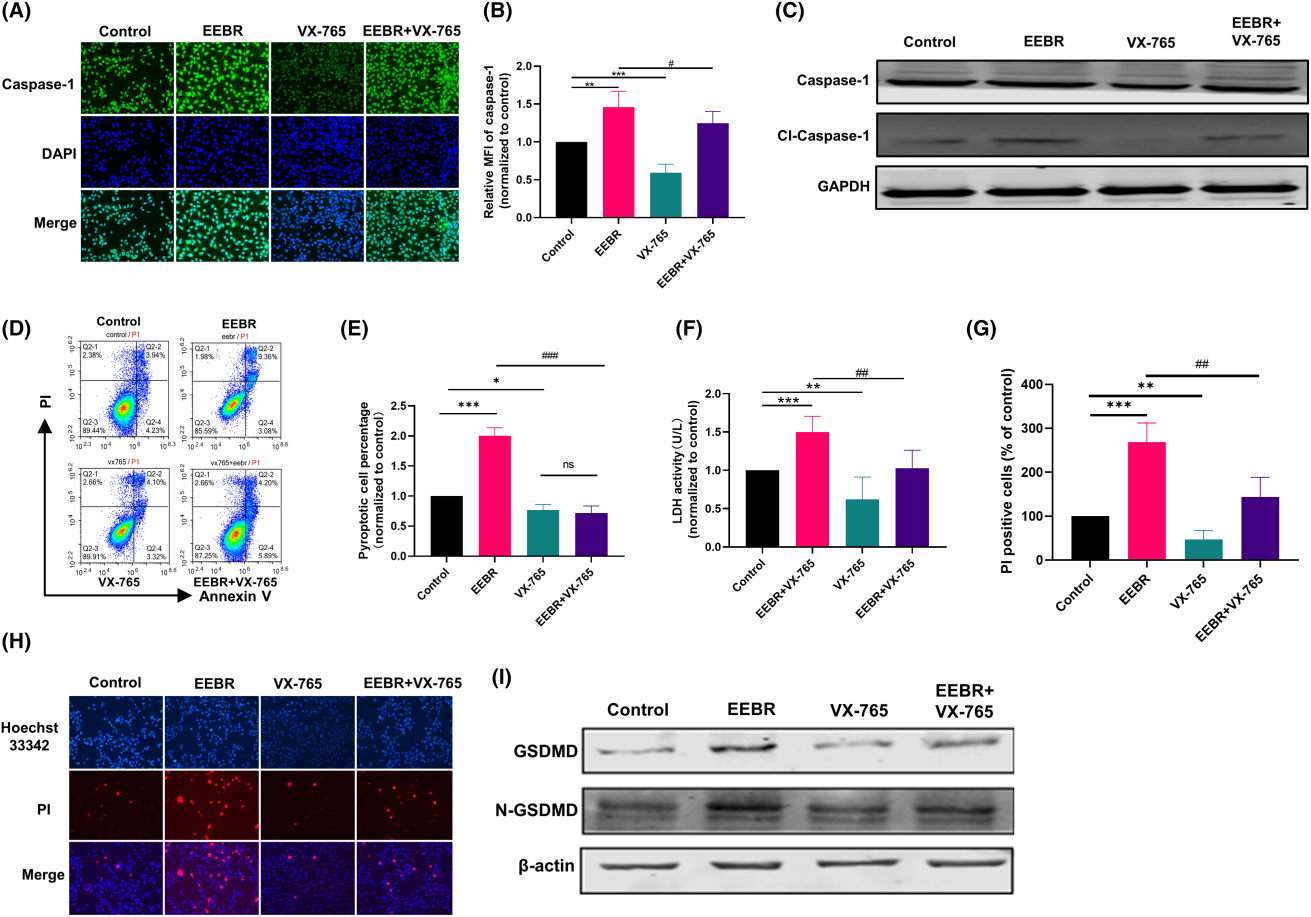
EEBR induces Caspase‐1‐dependent GSDMD cleavage. (A) Representative images of Caspase‐1 immunofluorescence. (B) Statistical plot of Caspase‐1 relative fluorescence intensity. (C) The expression of Caspase‐1 and cleaved‐Caspase‐1 in A549 cells with EEBR or VX‐765 determined by western blot analysis. (D, E) Flow cytometry analysis of Annexin V/PI double staining and pyroptosis cells in the Q2 area. (F) Effects of different treatments on induced LDH activity. (G) Statistical plot of the percentage of PI‐positive cells. (H) Images of Heochst 33342/PI double fluorescence staining. (I) Protein expression levels of GSDMD and N‐GSDMD. The data are presented as the mean ± SEM. *p < 0.05; **p < 0.01; ***p < 0.001 versus Control. ^#^ *p* < 0.05; ^##^ *p* < 0.01; ^###^ *p* < 0.001 versus EEBR; *n* = 4.

To investigate whether EEBR interacts through a Caspase‐1‐dependent manner, we added the Caspase‐1‐specific inhibitor VX‐765 to the cell culture medium in combination with EEBR. Immunofluorescence analysis showed that the combination of VX‐765 and EEBR significantly decreased the expression of Caspase‐1 induced by EEBR in A549 cells, compared to the EEBR‐only group (Figure 3A,B). Although immunoblotting experiments showed no significant change in the expression of Caspase‐1 in A549 treated with EEBR, the level of cleaved Caspase‐1 was partially reversed by VX‐765 (Figure 3C). Furthermore, the percentage of Annexin V^+^/PI^+^ cells was reduced (Figure 3D,E), and the combination of VX‐765 with EEBR reversed EEBR‐induced LDH release (Figure 3F) and the proportion of PI uptake in A549 cells (Figure 3G,H). Additionally, the presence of VX‐765 restored the protein levels of EEBR‐upregulated GSDMD and NT‐GSDMD (Figure 3I). In summary, these data suggest that EEBR activates Caspase‐1 to induce pyroptosis in NSCLC cells.

### 3.4 EEBR regulates the activation of NLRP3 and NF‐κB

**FIGURE 4:**
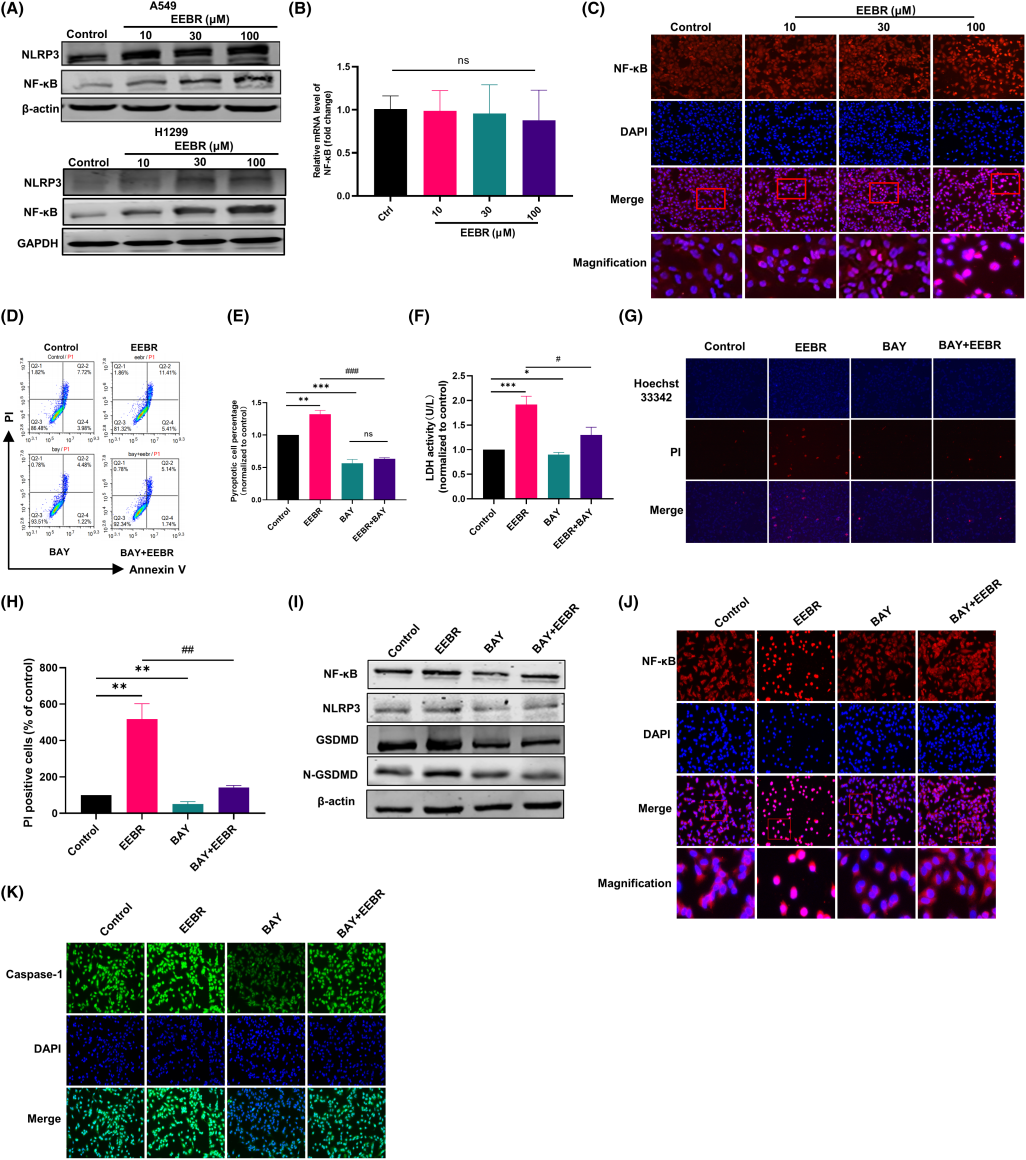
EEBR induces pyroptosis in a NF‐κB‐dependent manner in NSCLC cells. (A) The expression of NLRP3 and NF‐κB in A549 and H1299 cells of various groups. (B) mRNA levels of NF‐κB. (C) The fluorescence intensity of NF‐κB in A549 cells. (D, E) Flow cytometry analysis of Annexin V/PI double staining and pyroptosis cells in the Q2 area. (F) Effects of different treatments on induced LDH activity. (G) Images of Heochst 33342/PI double fluorescence staining. (H) Statistical plot of the percentage of PI‐positive cells. (I) Protein expression of NF‐κB, NLRP3, GSDMD and N‐GSDMD in A549 cells after treatments with varying agents. (J) Immunofluorescence representation of the distribution of NF‐κB in A549 cells. Red fluorescence represents the total expression level of NF‐κB, and blue staining indicates the cell nucleus. Addition of EEBR promotes the nuclear translocation of NF‐κB (second panel), while BAY can reverse the effect of EEBR, causing NF‐κB to redistribute back to the cytoplasm (fourth panel). (K) Immunofluorescence staining of Caspase‐1 distribution in cells. The addition of EEBR resulted in a significant increase in Caspase‐1 expression (second panel), while co‐treatment with BAY reversed the effect of EEBR, leading to a substantial reduction in Caspase‐1 expression (fourth panel). The data are presented as the mean ± SEM. *p < 0.05; **p < 0.01; ***p < 0.001 versus Control. ^#^ *p* < 0.05; ^##^ *p* < 0.01; ^###^ *p* < 0.001 versus EEBR; *n* = 4.

The assembly of pro‐Caspase‐1, NLRP3 and ASC forms the NLRP3 inflammasome, which subsequently activate Caspase‐1.^9^ We, therefore, investigated the effect of EEBR on the NLRP3 inflammasome. EEBR treatment for 24 h significantly increased the expression of NLRP3, compared to the control group, in a dose‐dependent manner (Figure 4A). The initiation step of NLRP3 inflammasome activation is the induction of NLRP3 transcription through activation of NF‐κB signalling.^17^ Although EEBR failed to affect the mRNA level of NF‐κB in A549 cells, we observed a dose‐dependent up‐regulation of the protein level of NF‐κB with increasing EEBR concentration (Figure 4A,B). Furthermore, we found that EEBR at 10, 30 and 100 μM significantly enhanced fluorescence intensity of NF‐κB and promoted NF‐κB entry into the nucleus (Figure 4C). These findings indicate that EEBR can activate NLRP3 and NF‐κB in a dose‐dependent manner.

### 3.5 EEBR induces pyroptosis in a NF‐κB‐dependent manner in NSCLC cells

To further confirm whether EEBR induces pyroptosis via NF‐κB, we administered BAY11‐7082 (BAY), an inhibitor of NF‐κB, in A549 cells, which is known to downregulate expression of NF‐κB by inhibiting TNF‐α‐induced phosphorylation of IκB‐α.^18^ As expected, co‐treatment of BAY with EEBR reversed the elevated ratio of Annexin V^+^/PI^+^ cells (Figure 4D,E) and LDH release induced by EEBR, compared with EEBR treatment alone (Figure 4F). Hoechst/PI double staining further confirmed that NF‐κB played an important role in EEBR‐induced pyroptosis (Figure 4G,H). Correspondingly, the elevated protein levels of NF‐κB, NLRP3 and GSDMD induced by EEBR were rectified with BAY (Figure 4I). The control group exhibited cytoplasmic and cell membrane localization of NF‐κB. After 24 h of treatment with EEBR, the translocation of NF‐κB towards the nucleus was increased. However, the translocation of NF‐κB to the nucleus was reversed after BAY was co‐treated with EEBR, and the fluorescence intensity of NF‐κB was significantly reduced in the BAY and EEBR co‐treatment group compared to the EEBR group (Figure 4J). The same trend was also observed for Caspase‐1 expression (Figure 4K). Therefore, our results suggest that EEBR triggers pyroptosis via the NF‐κB‐NLRP3‐GSDMD pathway.

### 3.6 EEBR suppresses the progression of NSCLC in vivo

**FIGURE 5:**
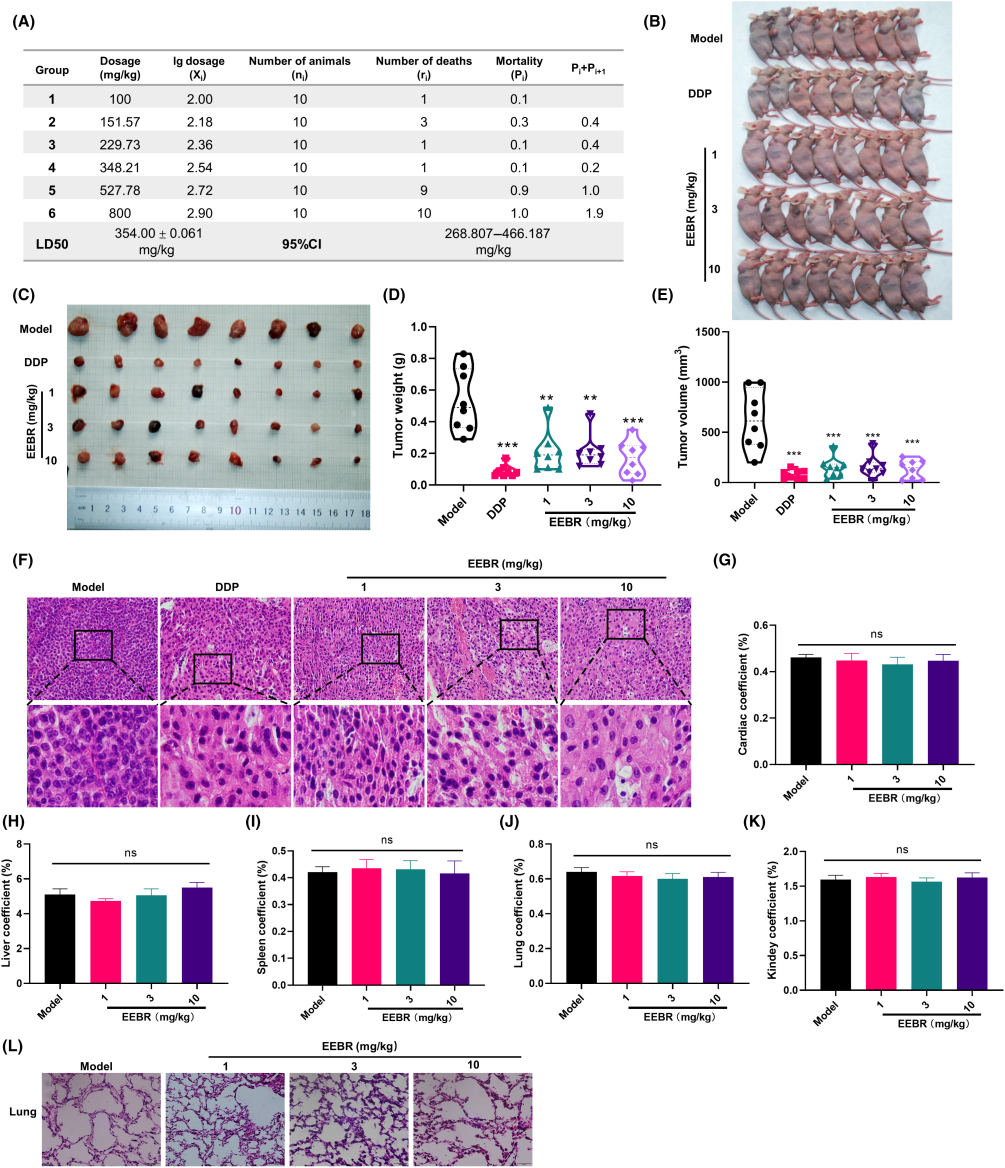
EEBR suppresses the progression of NSCLC in vivo. (A) Data related to LD50 calculation for EEBR. (B) After 28 days of treatment with EEBR and DDP, the mice bearing tumours were sacrificed. (C) Representative figures of tumour tissues. (D, E) Effects of EEBR on tumour weight (D) and volume (E) in each group. The data are presented as the mean ± SEM. *p < 0.05; **p < 0.01; ***p < 0.001versus Model; *n* = 8. (F) HE staining of tumour tissues (original magnification, 200×); *n* = 4. (G–K) Heart, liver, spleen, lung and kidney coefficients in EEBR‐treated mice (*n* = 5). (L) Representative diagram of pathological staining of the lungs of mice.

In order to evaluate the safety of EEBR, an acute toxicity assay was performed, employing the Koch method to determine the LD50 of EEBR as 354.00 ± 0.061 mg/kg (95% confidence interval 268.807–466.187 mg/kg, Figure 5A). To further investigate the effect of EEBR on tumour growth by inducing pyroptosis, we established a NSCLC xenograft model by subcutaneous injection of A549 cells and treating them with EEBR at doses of 1, 3 and 10 mg/kg. As shown in Figure 5B–E, EEBR treatment inhibited tumour growth in a dose‐dependent manner. Furthermore, at a dose of 10 mg/kg, EEBR inhibited tumour progression similar to the positive control drug, DDP. As a result, EEBR decreased the size and weight of tumours. Haematoxylin and eosin staining performed on tumour tissue sections revealed that tumour cells in the model group were morphologically diverse with enlarged nuclei and tightly arranged cells, while the EEBR group exhibited significantly reduced and sparsely arranged cells (Figure 5F). Additionally, our findings suggest that EEBR has minimal impact on organ coefficients and does not result in any pathological changes in the lungs or kidneys of tumour‐bearing mice (Figure 5G–L). These results further suggest that EEBR is capable of inhibiting tumour growth in vivo.

### 3.7 EEBR activates NF‐κB and induces Caspase‐1‐dependent pyroptosis in vivo

**FIGURE 6:**
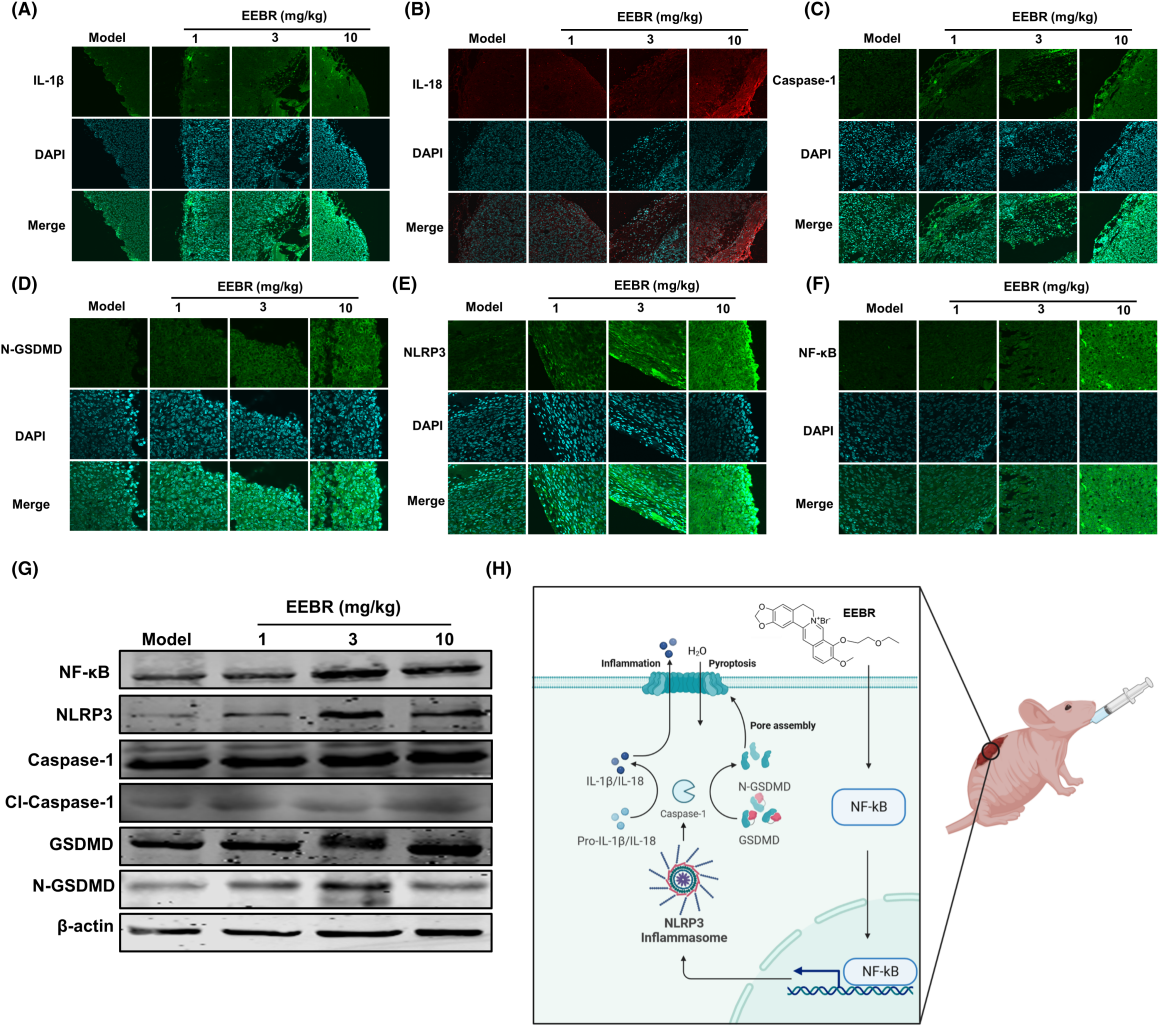
EEBR activates NF‐κB and induces Caspase‐1‐dependent pyroptosis in vivo. (A–F) Representative images and relative fluorescence intensity of IL‐1β, IL‐18, Caspase‐1, N‐GSDMD, NLRP3, NF‐κB in tumour tissue sections. (G) Protein levels of NF‐κB, NLRP3, Caspase‐1, cleaved Caspase‐1, GSDMD and N‐GSDMD in tumour tissues. (H) Schematic diagram of the proposed molecular mechanisms by which EEBR‐induced Caspase‐1‐dependent pyroptosis in non‐small cell lung cancer. The data are presented as the mean ± SEM. *p < 0.05; **p < 0.01; ***p < 0.001 versus Model; *n* = 4.

In order to investigate whether EEBR inhibits NSCLC progression in vivo via the NF‐κB‐NLRP3‐GSDMD pathway by promoting pyroptosis, we evaluated the levels of IL‐1β and IL‐18 in tumour tissues. Our results indicated that treatment with EEBR significantly increased the fluorescence intensity of IL‐1β (Figure 6A), IL‐18 (Figure 6B), Caspase‐1 (Figure 6C), N‐GSDMD (Figure 6D), NLRP3 (Figure 6E), NF‐κB (Figure 6F) compared to the model group. These results suggest that EEBR may promote the secretion of inflammatory factors, such as IL‐1β and IL‐18, xenografts. This is consistent with previous studies on pyroptosis, which is characterized by the release of pro‐inflammatory cytokines during cell death. Additionally, EEBR administration upregulated the expression of NF‐κB, NLRP3, cleaved Caspase‐1, GSDMD and N‐GSDMD (Figure 6G). Taken together, these findings suggest that EEBR may interfere with NSCLC progression in vivo by activating the NF‐κB‐NLRP3‐GSDMD pathway and facilitating pyroptosis. This approach could potentially provide a promising therapeutic strategy for the treatment of NSCLC (Figure 6H).

## 4 DISCUSSION

The incorporation of quaternary nitrogen into the alkaloid structural backbone has been found to be a significant anti‐cancer attribute.^11^ EEBR was created by synthesizing multiple factors, including its solubility in aqueous environments. In silico drug‐likeness assessment was carried out on EEBR using ChemDraw Ultra software, which included the calculation of Lipinski's parameters that can predict synthetic compound oral bioavailability and membrane permeability. Lipinski's rules are important because they describe the molecular properties associated with human pharmacokinetics, such as absorption, distribution, metabolism and excretion (ADME). The oil–water partition coefficient (Log*P*) is an essential factor reflecting the water (lipid) solubility of compounds, where high log*P* compounds have poor water solubility, and low log*P* compounds have poor permeability.^19^ A high Log*P* might limit the absorption properties of these compounds in the biological system due to poor solubility in water. EEBR's Log*P* is −0.049, ensuring its good receptor affinity. Additional molecular descriptors, such as topological polar surface area (TPSA), were calculated to further evaluate EEBR.^20^ According to Veber's rules, EEBR's TPSA value of 49.16 is less than 140, indicating potential intestinal absorption, bioavailability and blood–brain barrier permeability (Figure S2A–D). Our study suggests that EEBR exhibits favourable bioavailability characteristics and good drug performance based on the above‐mentioned parameters.

In terminal stage, cancer patients often suffer from extensive dissemination and metastasis, which may lead to the failure of vital organs and death. Hence a reliable anti‐cancer agent must have the ability of inhibiting tumour cell proliferation, advanced aggressiveness and tendency to metastasis. We investigated the impact of EEBR on NSCLC both in vitro and in vivo. The results demonstrated that EEBR effectively suppresses A549 and H1299 cell viability, proliferation, migration and invasion. In addition, EEBR treatment resulted in a significant reduction in tumour size and mass in our xenograft experiments compared to the model group. Taken together, these results suggest that administration of EEBR may serve as a promising strategy for inhibiting the progression of NSCLC.

Pyroptosis is characterized by progressive cellular swelling culminating in the rupture of the cell membrane, thereby triggering the release of intracellular contents and eliciting a robust inflammatory reaction. Pyroptosis serves as a significant innate immune response, crucial in countering infections and endogenous perilous cues. Distinguished from apoptosis, pyroptosis exhibits accelerated kinetics and concomitantly releases substantial quantities of pro‐inflammatory mediators. Pyroptosis is a mechanism involved in the progression of tumorigenesis and tightly associated with carcinogenesis and possibly applied in the inhibition of tumour.^21^ It has previously been assumed that many conventional chemotherapeutic agents used for NSCLC, such as gefitinib, cisplatin, paclitaxel, erlotinib and crizotinib, induce apoptosis and autophagic death.^22^, ^23^, ^24^, ^25^ However, this conclusion seems to be contradictory to the results reported in a few recent works. For instance, in addition to the typical apoptotic features, pyroptosis phenomena such as cell swelling, loss of cell membrane integrity and NT‐GSDME fragment generation were also observed, and the efficacy was more apparent with cisplatin treatment, indicating that paclitaxel and cisplatin both induce apoptosis and pyroptosis in lung cancer cells.^26^, ^27^ It has also been shown that the platinum‐based third‐generation agent Lobaplatin eradicates tumour cells through GSDME‐dependent pyroptosis.^14^ In addition, other natural compounds such as polyphyllin^28^ and cucurbitacin B (CuB) promoted the pyroptosis of tumour cells in a GSDMD‐dependent manner.^29^ EEBR is synthesized based on the natural compound berberrubine, so it may also tend to accelerate the pyroptosis of tumour cells. We investigated the morphological profile of A549 exposed to EEBR for the presence of pyroptosis cells, and electron scanning microscopy found that the EEBR‐ treatment caused cell membranes rupture with increased permeability to dyes, resulting in increased percentage of PI‐positive cells. Moreover, the osmotic lysis of cells also resulted in additional release of LDH.

NF‐κB hyperactivation induces elevated pro‐inflammatory cytokines, chemokines, immune receptors, adhesion molecules and is strongly associated with pyroptosis. In this study, we found that EEBR promoted nuclear translocation and expression of NF‐κB and activated NLRP3 in A549 and tumour tissues, while BAY treatment reduced the proportion of PI‐positive cells as well as the release of LDH to reverse the EEBR‐induced pyroptosis. Activation of NLRP3 inflammasome causes Caspase‐1 cleavage and secretion of IL‐1β and IL‐18.^30^ The expression of NLRP3, Caspase‐1 and secretion of IL‐18 and IL‐1β were upregulated in EEBR‐treated NSCLC cells and tissues, and the EEBR‐activated NLRP3‐mediated inflammatory response was ameliorated by BAY pretreatment. The acute activation of Caspase‐1 upon EEBR treatment enables cleavage of GSDMD, resulting in higher expression of GSDMD and NT‐GSDMD in NSCLC cells and tissues and oligomerization of NT‐GSDMD into pores on the membrane, ultimately coercing pyroptosis.^6^ In contrast, VX‐765 decreased the percentage of PI‐positive cells and the release of LDH, reversing EEBR‐induced pyroptosis and reduced expression of Caspase‐1 further contributed to less cleavage of N‐GSDMD. The potential mechanism underlying the promotion of pyroptosis and release of inflammatory mediators by EEBR through the regulation of the NF‐κB/NLRP3/Caspase‐1/GSDMD pathway has been proposed. It is pertinent to consider that the nuclear translocation of NF‐κB is associated with cell apoptosis,^31^ suggesting that EEBR may exert an influence on inducing apoptosis in lung cancer cells.

## 5 CONCLUSION

In conclusion, our results demonstrate that EEBR inhibits lung carcinogenesis and development. The underlying mechanism is likely that EEBR produces pyroptosis and induces the secretion of inflammatory cytokines by initiating the NF‐κB/NLRP3/Caspase‐1/GSDMD pathway in NSCLC. Therefore, EEBR might be a new drug candidate for NSCLC. Our research provides a novel strategy for cancer treatment by targeting cancer cell pyroptosis.

## AUTHOR CONTRIBUTIONS

**Xin Zhao:** Conceptualization (equal); data curation (equal); funding acquisition (equal). **Chao Chen:** Investigation (equal); writing – original draft (equal). **Weina Han:** Methodology (equal). **Min Liang:** Data curation (equal); investigation (equal). **Yuanyuan Cheng:** Investigation (equal); visualization (equal). **Yingjie Chen:** Investigation (equal); software (equal). **Defeng Pang:** Formal analysis (equal); investigation (equal). **Haoqi Lei:** Investigation (equal); visualization (equal). **Xuefei Feng:** Formal analysis (equal); investigation (equal). **Shifeng Cao:** Software (equal). **Zhixiong Li:** Methodology (equal). **Jinhui Wang:** Conceptualization (equal). **Yan Zhang:** Project administration (equal); writing – review and editing (equal). **Baofeng Yang:** Conceptualization (equal); project administration (equal).

## CONFLICT OF INTEREST STATEMENT

The authors declared no potential conflicts of interest to this article's research, authorship and publication.

## Supporting information


Figures S1–S2

## Data Availability

The data that support the findings of this study are available on request from the corresponding author. The data are not publicly available due to privacy or ethical restrictions.
