# Supplementary material for: EEBR induces Caspase‐1‐dependent pyroptosis through the NF‐κB/NLRP3 signalling cascade in non‐small cell lung cancer
Source: J Cell Mol Med. 2024 Jan 12;28(3):e18094. doi: 10.1111/jcmm.18094 (PMC10844718; doi:10.1111/jcmm.18094)
Supplement: Supplementary file 1 — Figures S1–S2 [file JCMM-28-e18094-s001.zip › Legends.docx]

**Figure S1. Short Tandem Repeat (STR) analysis to confirm the identity of A549 and H1299 cell lines.** (A) STR analysis results for the A549 cell line indicated a 100% match between the DNA profile of the tested cells and the reference profile in the cell bank. The verified name of the cell line was A549. (B) STR analysis results for the H1299 cell line demonstrated a 98.04% match between the DNA profile of the tested cells and the reference profile in the cell bank. The validated name of the cell line was H1299.

**Figure S2. Chemical synthesis and characterization of EEBR.** (A) In silico drug-likeness assessment was carried out on EEBR using ChemDraw Ultra software, which included calculation of chemical formula, molecular weight, tPSA, log P and CMR. (B) Synthesis rout of EEBR. (C-D) Mass spectrometric characterization of EEBR.
