# Supplementary figures and images for: EEBR induces Caspase‐1‐dependent pyroptosis through the NF‐κB/NLRP3 signalling cascade in non‐small cell lung cancer
Source: J Cell Mol Med. 2024 Jan 12;28(3):e18094. doi: 10.1111/jcmm.18094 (PMC10844718; doi:10.1111/jcmm.18094)

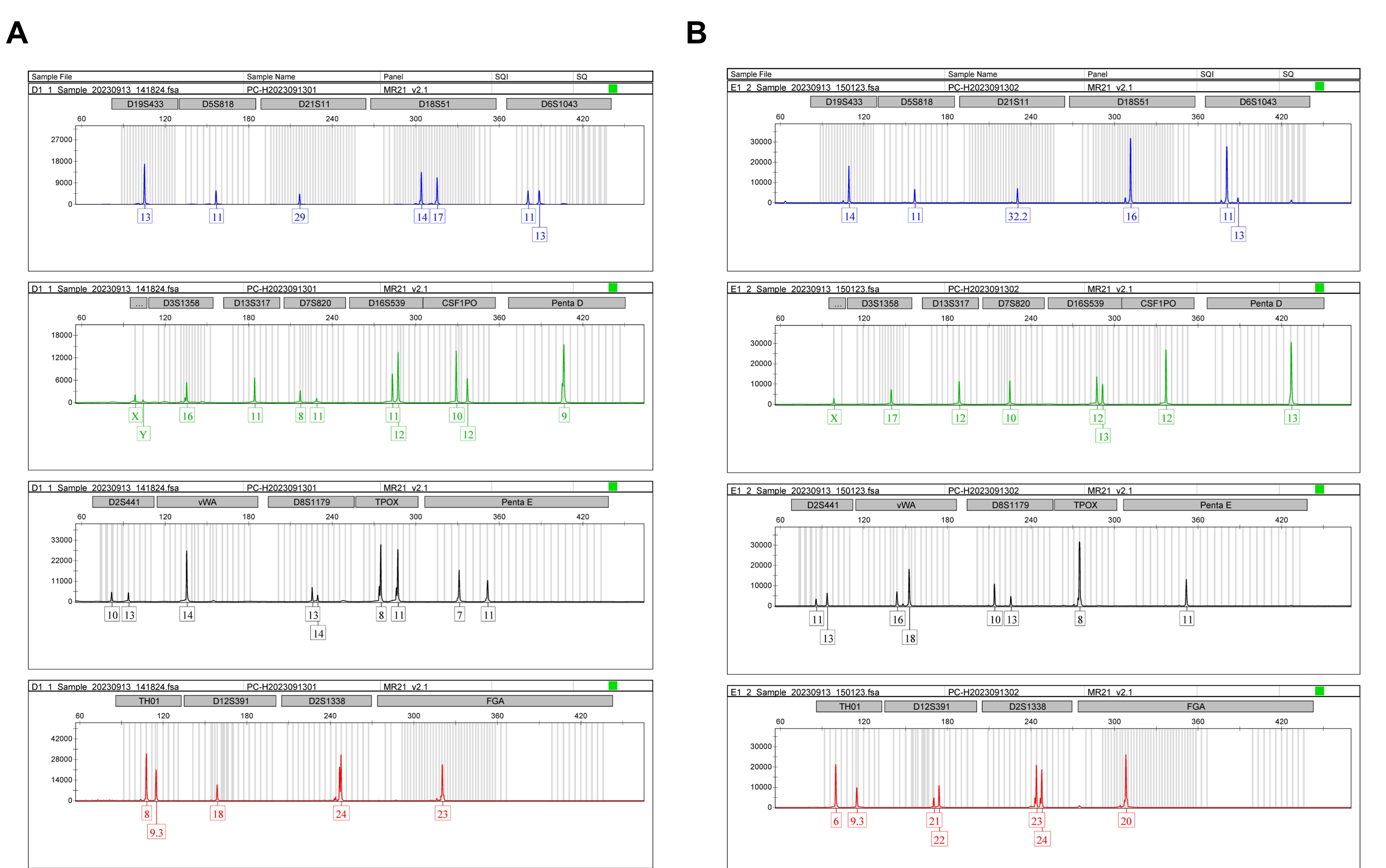

Supplement: Supplementary file 1 — Figures S1–S2 [file JCMM-28-e18094-s001.zip › jcmm18094-sup-0001-FigureS1.tif]

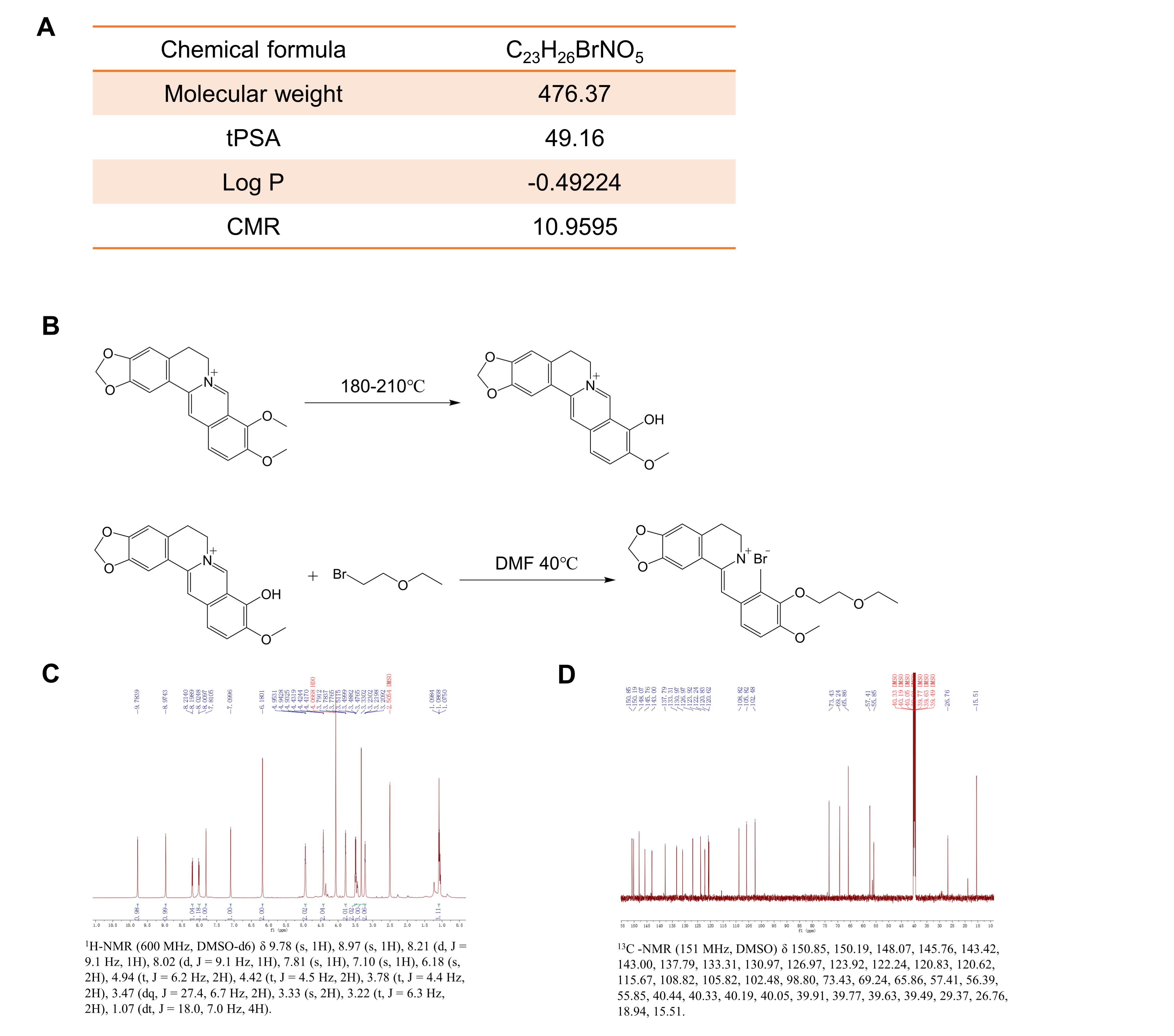

Supplement: Supplementary file 1 — Figures S1–S2 [file JCMM-28-e18094-s001.zip › jcmm18094-sup-0002-FigureS2.tif]
